# LncRNA NDUFA6-DT: A Comprehensive Analysis of a Potential LncRNA Biomarker and Its Regulatory Mechanisms in Gliomas

**DOI:** 10.3390/genes15040483

**Published:** 2024-04-11

**Authors:** Ruiting Huang, Ying Kong, Zhiqing Luo, Quhuan Li

**Affiliations:** 1 School of Biology and Biological Engineering, South China University of Technology, Guangzhou 510006, China; 202121050567@mail.scut.edu.cn (R.H.); 202020148659@mail.scut.edu.cn (Y.K.); 202121050573@mail.scut.edu.cn (Z.L.); 2 Guangdong Provincial Engineering and Technology Research Center of Biopharmaceuticals, South China University of Technology, Guangzhou 510006, China

**Keywords:** glioma, NDUFA6-DT, lncRNA, biomarker, prognostic factor, regulatory networks

## Abstract

Gliomas are the most prevalent primary malignant tumors affecting the brain, with high recurrence and mortality rates. Accurate diagnoses and effective treatment challenges persist, emphasizing the need for identifying new biomarkers to guide clinical decisions. Long noncoding RNAs (lncRNAs) hold potential as diagnostic and therapeutic biomarkers in cancer. However, only a limited subset of lncRNAs in gliomas have been explored. Therefore, this study aims to identify lncRNA signatures applicable to patients with gliomas across all grades and explore their clinical significance and potential biological mechanisms. Data used in this study were obtained from TCGA, CGGA, and GEO datasets to identify key lncRNA signatures in gliomas through differential and survival analyses and machine learning algorithms. We examined their associations with the clinical characteristics, gene mutations, diagnosis, and prognosis of gliomas. Functional enrichment analysis was employed to elucidate the potential biological mechanisms associated with these significant lncRNA signatures. We explored competing endogenous RNA (ceRNA) regulatory networks. We found that NDUFA6-DT emerged as a significant lncRNA signature in gliomas, with reduced NDUFA6-DT expression associated with a worse prognosis in gliomas. Nomogram analysis incorporating NDUFA6-DT expression levels exhibited excellent prognostic and predictive capabilities. Functional annotation suggested that NDUFA6-DT might influence immunological responses and synaptic transmission, potentially modifying glioma initiation and progression. The associated ceRNA network revealed the possible presence of the NDUFA6-DT-miR-455-3p-YWHAH/YWHAG axis in low-grade glioma (LGG) and glioblastoma multiforme (GBM), regulating the PI3K-AKT signaling pathway and influencing glioma cell survival and apoptosis. We believe that NDUFA6-DT is a novel lncRNA linked to glioma diagnosis and prognosis, potentially becoming a pivotal biomarker for glioma.

## 1. Introduction

Gliomas stand as the predominant primary intracranial tumor within the central nervous system, constituting 75% of malignant brain tumors in adults. This condition is characterized by high malignancy, resulting in a poor prognosis. Gliomas exhibit a tendency for frequent recurrence, are drug-resistant, and pose significant challenges in achieving a cure. Globally, gliomas have an incidence rate ranging from 4.67 to 5.73/100,000 individuals, and the 5-year overall survival rate is <35% [1,2]. The 2021 WHO classification categorizes gliomas into astrocytomas, oligodendrogliomas, and glioblastoma multiforme (GBM), with astrocytomas and oligodendrogliomas falling under the designation of low-grade glioma (LGG) [3]. The standard treatment protocol for gliomas includes surgical excision of the tumor, radiotherapy, and chemotherapy [1]. However, survival rates for patients are significantly low; in particular, 70% of patients with LGG progress to GBM, and the 5-year survival rate for GBM is <5% [4]. Incorporating histopathological and molecular features into the new classification significantly improves the objectivity and accuracy of diagnostics [3]. However, the complex pathogenesis and molecular heterogeneity of gliomas play a role in the suboptimal therapeutic outcomes. Therefore, novel and reliable molecular targets or prognostic markers are urgently required to improve diagnostic and therapeutic effectiveness regarding gliomas.

Long noncoding RNAs (lncRNAs) are RNA transcripts that exceed 200 nucleotides in length but do not encode proteins. Despite being initially considered as transcriptional noise [5,6], accumulating evidence in recent years underscores the role of lncRNAs as regulatory factors in various cellular processes. Moreover, they play a role in tumor development and metastasis [7,8]. They may serve as prognostic indicators or targets for glioma treatment [9,10]. Within gliomas, specific lncRNAs, such as HOXA11-AS [11] and LPP-AS2 [12], actively contribute to cell proliferation, migration, and drug resistance through diverse mechanisms. A correlation has been observed between the decreased expression of lncRNA CASC2, elevated levels of miR-193a-5p, and reduced mTOR expression, resulting in protective autophagy and resistance to temozolomide [13]. However, only a limited subset of lncRNAs in gliomas have received attention. Consequently, exploring the functional and regulatory mechanisms of these valuable lncRNAs is crucial for gaining significant diagnostic and therapeutic insights into the disease. The identification of novel lncRNA biomarkers and a comprehensive exploration of their roles and regulatory mechanisms in gliomas hold significant implications for disease diagnosis and treatment. Therefore, this study aims to identify lncRNA biomarkers applicable across all glioma grades to enhance glioma diagnosis, prognosis, and personalized treatment. This research is motivated by the suboptimal outcomes observed in current glioma diagnostic and therapeutic approaches, as well as the emerging recognition of lncRNAs’ pivotal regulatory roles in gliomas.

**Figure 1:**
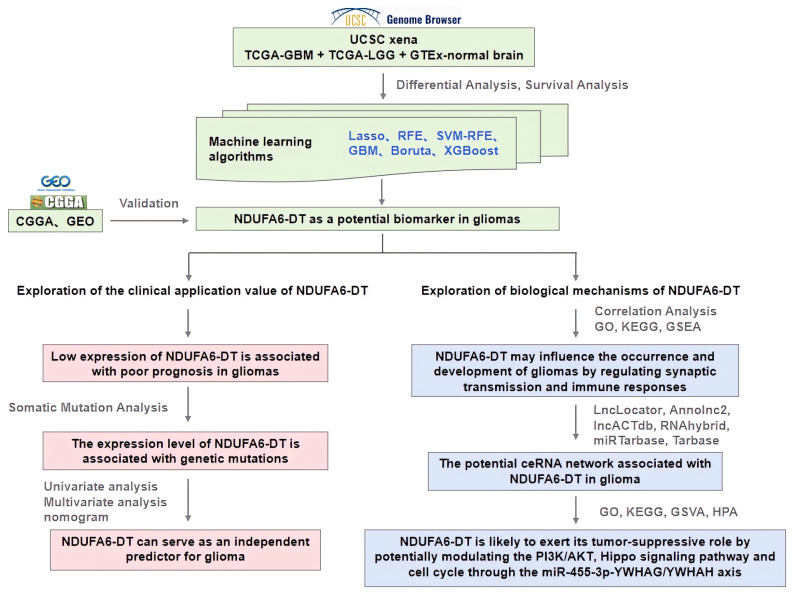
Analytical flowchart for this study. TCGA-LGG: low-grade glioma; TCGA-GBM: glioblastoma multiforme; Lasso: least absolute shrinkage and selection operator; RFE: recursive feature elimination; SVM-RFE: support vector machine–recursive feature elimination; GBM: gradient-boosting machine; XGBoost: extreme gradient boosting; GO: Gene Ontology; KEGG: Kyoto Encyclopedia of Genes and Genomes; GSEA: gene set enrichment analysis; ceRNA: competing endogenous RNA.

The present study performed a bioinformatic analysis of glioma samples, including LGG and GBM, to investigate lncRNA biomarkers relevant to patients at all stages of glioma and elucidate their clinical significance and underlying mechanisms of action (Figure 1). Initially, significant lncRNA signatures were identified through differential and survival analyses, as well as various machine learning algorithms. Subsequently, the expression patterns of NDUFA6-DT were examined in relation to clinical characteristics and the genome, while evaluating the clinical significance for glioma prognosis. Finally, we reveal the potential biological functions and ceRNA networks of NDUFA6-DT in gliomas.

## 2. Materials and Methods

### 2.1. Data Collection and Preprocessing

The UCSC Xena platform (http://xena.ucsc.edu/, accessed on 4 September 2023) [14] was utilized to download data on TCGA-LGG and TCGA-GBM, including RNA-seq gene expression and clinical survival files, and RNA-seq gene expression files of GTEx normal brain tissues. The data were merged. Subsequently, batch effects were eliminated and normalization was performed using the R package limma, resulting in the creation of a normalized expression matrix. The Ensembl database (https://asia.ensembl.org/index.html, accessed on 4 September 2023) was employed for gene re-annotation to extract mRNA and lncRNA expression matrices. Validation was conducted using the CGGA and GEO datasets. Glioma gene expression files and clinical data were acquired from the CGGA database (http://www.cgga.org.cn/, accessed on 4 September 2023) [15], comprising the mRNAseq_693 and mRNAseq_325 cohorts. Batch effects were eliminated using the R package sva, followed by data integration. Three microarray datasets, namely GSE108474 [16], GSE90603 [17], and GSE25631 [18], were obtained from the GEO database (https://www.ncbi.nlm.nih.gov/geo/, accessed on 4 September 2023). Table 1 summarizes the datasets.

### 2.2. Identification of Differentially Expressed Genes

We conducted differential expression analysis of lncRNAs using the DESeq2 package [19]. The criteria for selecting differentially expressed lncRNAs (DElncRNAs) were |log2FC| ≥ 2 and padj < 0.01. For differentially expressed mRNAs (DEmRNAs), the criteria were |log2FC| ≥ 1 and padj < 0.01. The ggplot2 package was employed to visualize multiple-group volcano graphs.

### 2.3. Selection and Validation of Key lncRNAs

To identify essential lncRNA signatures in gliomas, we employed the R package ggvenn to visualize the common differentially expressed lncRNAs (co-DElncRNAs) present in LGG and GBM. Subsequently, we utilized the survival package for survival analysis of co-DElncRNAs, employing the log-rank test to assess survival differences between groups with high and low expressions (*p* < 0.05 denotes statistical significance). To identify co-DElncRNAs with significant survival implications in LGG and GBM, we utilized an online tool (https://www.omicshare.com/tools/, accessed on 4 September 2023) to construct a Venn diagram. Subsequently, we employed various R packages including glmnet, Caret, randomForest, e1071, Boruta, GBM, and XGBoost to implement the least absolute shrinkage and selection operator (Lasso), recursive feature elimination (RFE), support vector machine–recursive feature elimination (SVM-RFE), Boruta, a gradient-boosting machine (GBM), and extreme gradient boosting (XGBoost). These approaches were employed to select essential lncRNA signatures. We identified overlapping signatures as potential hub lncRNAs in gliomas through the application of six machine learning algorithms. The Survminer package was used to display potential hub lncRNAs based on the Kaplan–Meier survival analysis. The pROC package was employed to calculate the area under the ROC curve, evaluating the sensitivity and specificity of the lncRNA signatures in diagnosing patients with gliomas.

### 2.4. Somatic Mutation Analysis

The maftools package was utilized to analyze somatic mutation data [20]. Following the calculation, we identified genes with the highest frequency of mutations and mutation types. The TMB values of patients were computed using the tmb function. Survival rates of patients with high and low mutation burdens were compared using survival analysis.

### 2.5. Construction and Validation of Nomogram

We performed univariate and multivariate Cox regression analyses to explore the potential utility of NDUFA6-DT as an independent prognostic factor for gliomas. Predictions for the overall survival rates of patients with gliomas at 1, 3, and 5 years were predicted by constructing a nomogram using the RMS package. We computed calibration curves to assess the degree of agreement between actual survival probability and nomogram predictions. The timeROC package was employed to generate ROC curves, evaluating the accuracy of 1-, 3-, and 5-year survival rate predictions. Decision Curve Analysis (DCA) was conducted using the rmda package to evaluate the effectiveness of the constructed nomogram for therapeutic decision making.

### 2.6. Pathway Enrichment and Functional Analysis

Based on NDUFA6-DT expression levels, we categorized the samples into high- and low-expression groups. Differential expression analysis was conducted through the limma package, employing the criteria of log2FC ≥ |0.5| and padj < 0.01. Subsequently, genes strongly associated with NDUFA6-DT (correlation > |0.3|, *p* < 0.01) were identified through Pearson’s correlation analysis. We identified overlapping genes between genes showing significant correlations and differentially expressed genes as co-expressed genes of NDUAF6-DT. This analysis aims to provide insights into its potential biological functions and pathways in gliomas. Gene Ontology (GO) and Kyoto Encyclopedia of Genes and Genomes (KEGG) [21] enrichment analyses were conducted on the genes co-expressed with NDUFA6-DT using the clusterProfiler package. We considered only functional categories or pathways (*p*.adjust < 0.05) as significant. Gene set enrichment analysis (GSEA) was performed using the MSigDB Hallmark gene set (https://www.gsea-msigdb.org/gsea/msigdb, accessed on 9 October 2023). Results were considered statistically significant if FDR < 0.25 and *p*.adjust < 0.05. The PathView package [22] was utilized to visualize the locations and expression levels of genes in specific pathways. The HPA database (https://www.proteinatlas.org/, accessed on 1 April 2024) was used to detect the protein expression of specific genes. The GSVA package was utilized to assess the activity scores of specific pathways in glioma patients.

### 2.7. Immune Infiltration Analysis

The estimate package was used to calculate the immunity score and stroma score of glioma patients. The ssGSEA algorithm was employed to assess differences in immune cell infiltration and immune function within the NDUFA6-DT expression group. Pearson’s correlation analysis was conducted to explore the association between NDUFA6-DT expression, immune cells, and immune function. The ggcor package was utilized to generate a correlation network graph.

### 2.8. Prediction of the ceRNA Network for NDUFA6-DT

We obtained sequence information for NDUFA6-DT from the NCBI for Biotechnology Information database (https://www.ncbi.nlm.nih.gov/, accessed on 6 November 2023). Subcellular localization was predicted using LncLocator (http://www.csbio.sjtu.edu.cn/bioinf/lncLocator/, accessed on 6 November 2023). Interactions between NDUFA6-DT and miRNAs were predicted using AnnoLnc2 (http://annolnc.gao-lab.org/, accessed on 6 November 2023) and lncACTdb (http://bio-bigdata. hrbmu.edu.cn/lncACTdb/, accessed on 6 November 2023). The minimal free energy (mfe) and binding sites between lncRNA and miRNA were predicted using RNAhybrid (https://bibiserv.cebitec.uni-bielefeld.de/rnahybrid, accessed on 6 November 2023). Downstream mRNAs for the identified miRNAs were predicted using Tarbase (https://bio.tools/tarbase, accessed on 6 November 2023) and miRTarbase (https://mirtarbase.cuhk.edu.cn/~miRTarBase/miRTarBase_2022/php/index.php, accessed on 6 November 2023). The visualization of the ceRNA network was performed using the ggalluvial package.

### 2.9. Statistical Analysis

All statistical analyses and calculations in this study were conducted using R version 4.2.2 and the corresponding packages. Differences across groups were assessed using Student’s *t*-tests, where significance was considered at * *p* < 0.05, ** *p* < 0.01, *** *p* < 0.001, and **** *p* < 0.0001.

## 3. Results

### 3.1. NDUFA6-DT: A Potential Key lncRNA Signature in Glioma Pathology

**Figure 2:**
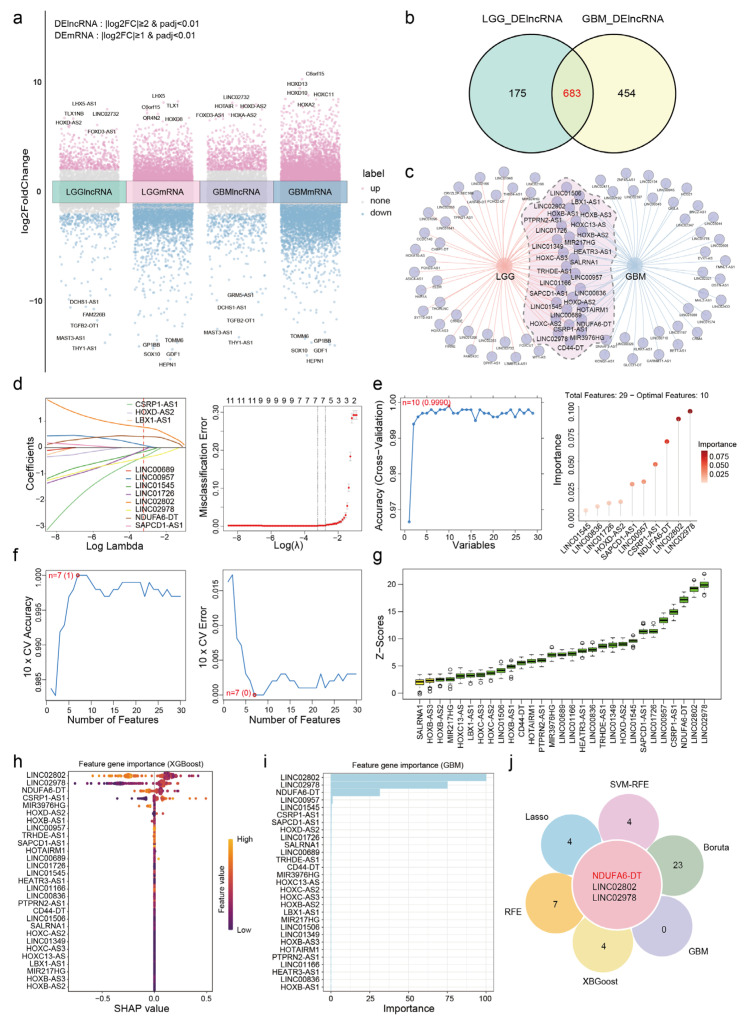
Comprehensive analysis reveals NDUFA6-DT as a potential lncRNA signature in gliomas. (**a**) Multiple volcano plots illustrate the differential expression of lncRNAs and mRNAs in LGG and GBM. Grey labels represent genes that are not differentially expressed, while pink and blue labels depict up- and downregulation, respectively. (**b**) The Venn diagram illustrates 683 overlapping DElncRNAs in LGG and GBM. (**c**) The network diagram illustrates 29 overlapping DElncRNAs with survival significance in LGG and GBM. (**d**) Lasso regression analysis exhibits seven crucial lncRNA signatures, showing the path plot of the regression coefficients and the curve for 10-fold cross-validation. (**e**) The RFE analysis shows 10 crucial lncRNA signatures, achieving the highest model accuracy with 10 variables, and shows the importance of the variables in the lollipop chart. (**f**) The SVM-RFE analysis reveals seven essential lncRNA signatures, resulting in the highest model accuracy and lowest error rate with 10 variables. (**g**–**i**) The importance of 29 lncRNAs in Boruta, GBM, and XGBoost algorithms, respectively. (**j**) Venn diagram illustrates the overlap of three core lncRNA signatures identified through six machine learning algorithms.

We conducted differential expression analysis to delineate distinctions between normal samples and those from patients with LGG or GBM. In LGG, we identified 858 DElncRNAs and 6767 DEmRNAs, while in GBM, the analysis revealed 1137 DElncRNAs and 8452 DEmRNAs (Figure 2a). The overlap of DElncRNAs between LGG and GBM revealed 683 shared DElncRNAs, potentially constituting crucial lncRNA signatures in gliomas (Figure 2b). Survival analysis revealed 29 DElncRNAs exhibiting survival differences between LGG and GBM (Figure 2c). We further investigated the 29 DElncRNAs using six machine learning algorithms and employing the Lasso logistic algorithm with a penalty parameter set at lambda.min = 0.03952; we identified seven significant lncRNAs (Figure 2d). The RFE algorithm, which achieved the highest accuracy (0.9990), revealed an optimal subset of the 10 crucial lncRNAs. The top three lncRNAs in the subset were LINC02978, LINC02802, and NDUFA6-DT (Figure 2e). The SVM-RFE algorithm, having the highest precision and lowest error rate, confirmed the identification of seven significant lncRNAs through 10-fold cross-validation (Figure 2f). We extracted essential parameters of the 29 lncRNA signatures using the Boruta, GBM, and XGBoost algorithms. The top three lncRNA signatures considered important by all three algorithms were LINC02978, LINC02802, and NDUFA6-DT (Figure 2g–i). Integrating the outcomes of these algorithms led to the identification of three crucial lncRNA signatures in glioma (Figure 2j).

We further evaluated the three lncRNA signatures based on their expression levels, survival differences, and AUC values. NDUFA6-DT and LINC02802 exhibited downregulation, while LINC02978 increased (Figure S1a). ROC curves demonstrated high diagnostic accuracy for these three lncRNA signatures in patients with gliomas (Figure S1b). Survival analysis revealed a significant difference in all three lncRNA signatures between LGG and GBM. However, only LINC02978 and NDUFA6-DT were found to be associated with prognosis (Figure S1c–h). We validated survival differences using the CGGA dataset as an external dataset. However, clinical information for LINC02978 was not available for inclusion in the survival analysis (Figure S1i–l). Only the survival difference in the NDUFA6-DT cells was validated using the CGGA dataset. Therefore, we hypothesize that NDUFA6-DT may serve as a crucial lncRNA signature in gliomas, warranting further investigation.

### 3.2. Low NDUFA6-DT Expression in Gliomas Correlates with Adverse Prognosis

**Figure 3:**
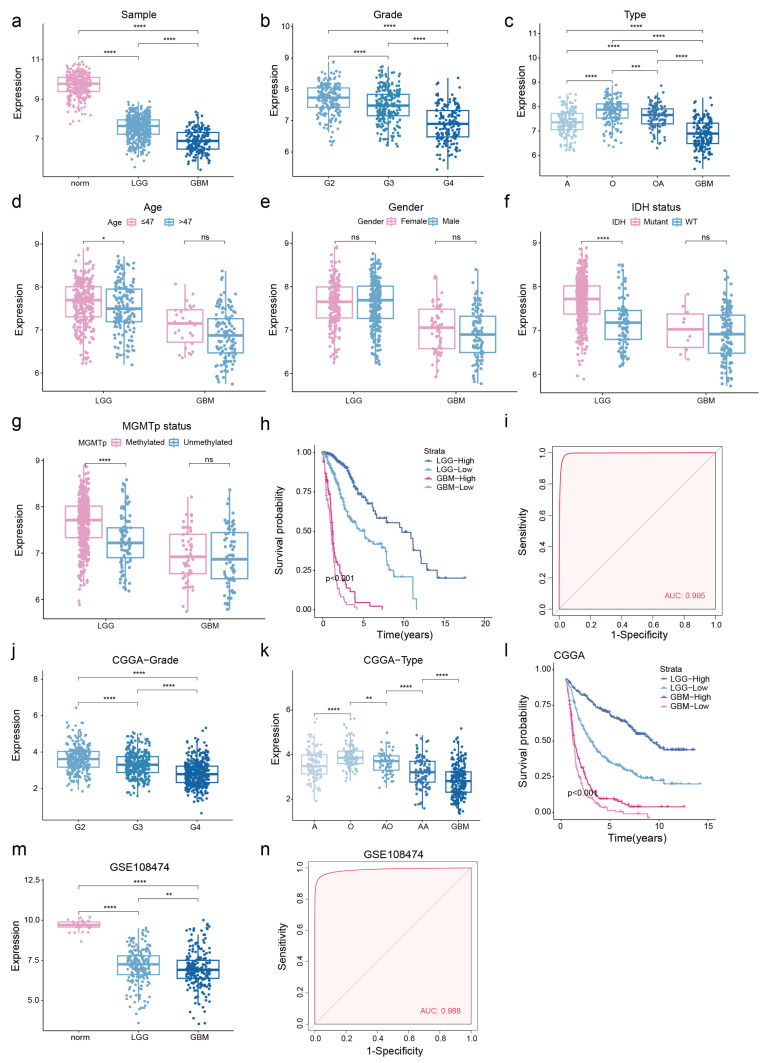
Investigating the potential significance of NDUFA6-DT in gliomas. (**a**) Illustration of NDUFA6-DT expression levels in normal, LGG, and GBM samples. (**b**–**g**) Analysis of NDUFA6-DT expression patterns in glioma samples based on factors including grades, histological types, age, sex, IDH mutation status, and MGMT methylation status. (**h**) Kaplan–Meier curves illustrating NDUFA6-DT in samples from patients with LGG and GBM. (**i**) ROC curve depicting NDUFA6-DT in gliomas. (**j**,**k**) Expression profile of NDUFA6-DT in glioma samples of various grades and histological types within CGGA. (**l**) Kaplan–Meier curves illustrating NDUFA6-DT in LGG and GBM samples from CGGA datasets. (**m**) Expression of NDUFA6-DT in normal, LGG, and GBM in the GSE108474 dataset. (**n**) ROC curve evaluating the performance of NDUFA6-DT in glioma within GSE108474. A, astrocytoma; O, oligoastrocytoma; OA, oligodendroglioma; GBM, glioblastoma; G2, Grade 2; G3, Grade3; G4, Grade 4. * *p* < 0.05, ** *p* < 0.01, *** *p* < 0.001, and **** *p* < 0.0001; ns, no significance.

To investigate the potential significance of NDUFA6-DT in gliomas, we analyzed its expression distribution profile in glioma clinicopathology. NDUFA6-DT exhibited downregulation in LGG and GBM compared to normal samples (Figure 3a). Furthermore, the expression showed a significant decrease with an increase in disease grade (Figure 3b). Statistically significant differences in NDUFA6-DT expression were observed among the different histological glioma types. In LGG, oligodendrogliomas exhibited a significantly higher expression level than oligoastrocytomas, followed by astrocytomas. The expression was lowest in GBM, characterized by the highest level of malignancy (Figure 3c). NDUFA6-DT exhibited differential expression between the younger (age ≤ 47) and older (age > 47) groups in LGG, displaying higher expression in the younger group, while no such difference was observed in GBM (Figure 3d). In LGG and GBM, no significant variations in NDUFA6-DT expression were observed based on sex (Figure 3e). The IDH mutant and MGMT promoter methylation groups in LGG exhibited significantly higher NDUFA6-DT expression than the IDH wild-type and MGMT promoter non-methylation groups. However, no differences in the expression of NDUFA6-DT were observed in GBM between the groups based on the molecular markers IDH and MGMT (Figure 3f,g). Survival curves showed an unfavorable prognosis in patients, with LGG and GBM patients exhibiting low NDUFA6-DT expression (Figure 3h). With an AUC score of 0.995, NDUFA6-DT exhibited excellent diagnostic accuracy for glioma disorders (Figure 3i). The expression and prognostic capacity of NDUFA6-DT in gliomas of various grades and histological types were validated using the CGGA dataset (Figure 3j–l). The low expression of NDUFA6-DT in diseased tissues and its high diagnostic accuracy for gliomas were confirmed using GSE108474 (Figure 3m,n). These findings emphasize the significance of NDUFA6-DT in the expression and prognosis of gliomas with different grades and histological types.

### 3.3. Correlation between NDUFA6-DT Expression and Genetic Mutations in Gliomas

**Figure 4:**
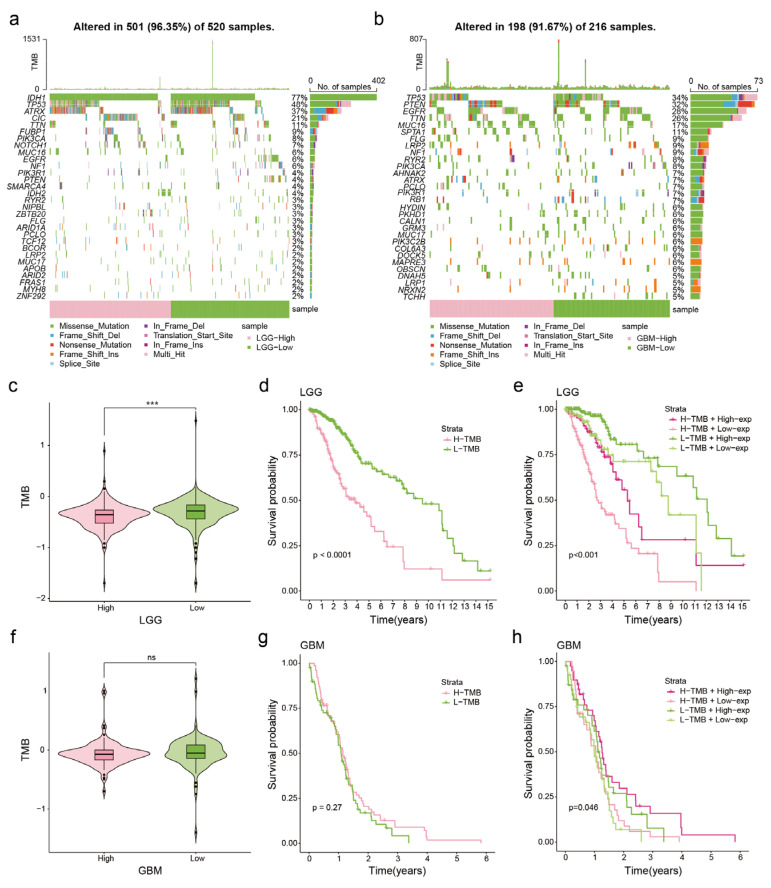
Association of NDUFA6-DT expression with glioma gene mutations. (**a**,**b**) Waterfall plots illustrate the top 30 genes and gene mutation types with the highest mutation frequencies in LGG and GBM, respectively. The pink region represents glioma samples with high NDUFA6-DT expression, while the green region denotes samples with low NDUFA6-DT expression. (**c**,**f**) Box plots illustrating the TMB values for the NDUFA6-DT expression group in LGG and GBM. (**d**,**g**) Kaplan–Meier curves illustrate TMB groups in LGG and GBM. (**e**,**h**) Kaplan–Meier curves display the combined NDUFA6-DT expression and TMB groups in LGG and GBM. TMB, tumor mutational burden. *** *p* < 0.001; ns, no significance.

Somatic mutation analysis was performed to explore genomic alterations linked to NDUFA6-DT expression. Utilizing NDUFA6-DT expression in LGG and GBM, waterfall plots were generated to illustrate the types and frequencies of mutations in the top 30 genes. Following the WHO standards for molecular pathology markers in gliomas, mutations in IDH1 and ATRX showed a better prognosis, while mutations in TP53, EGFR, and PTEN were associated with a poorer prognosis [23]. Patients with low expression of NDUFA6-DT exhibited a reduced frequency of IDH1 mutations and a higher prevalence of TP53 mutations than patients with high expression in LGG (Figure 4a). In GBM, the group with low NDUFA6-DT expression demonstrated a higher frequency of TP53 mutations. In contrast, the high- and low-expression groups showed comparable mutation frequencies for EGFR and PTEN (Figure 4b).

We examined the relationship between NDUFA6-DT expression and tumor mutational burden (TMB). The findings revealed that the group with decreased NDUFA6-DT expression exhibited increased TMB in LGG (Figure 4c), while in GBM, TMB did not show a significant difference based on the NDUFA6-DT expression group (Figure 4f). Based on the analysis of survival rates, patients with LGG with higher TMB had a shorter survival period than those with lower TMB (Figure 4d). However, TMB did not significantly affect the survival prognosis of patients with GBM (Figure 4g). Upon stratifying patients with glioma into four groups based on both NDUFA6-DT expression and TMB, we observed that in LGG, those with high NDUFA6-DT expression and low TMB exhibited the most significant survival advantage (Figure 4e). In GBM, patients with high NDUFA6-DT expression and TMB exhibited the best survival advantage (Figure 4h). These findings show the correlation between NDUFA6-DT expression and genetic changes in gliomas.

### 3.4. NDUFA6-DT: An Independent Prognostic Factor and Protective Element in Gliomas

**Figure 5:**
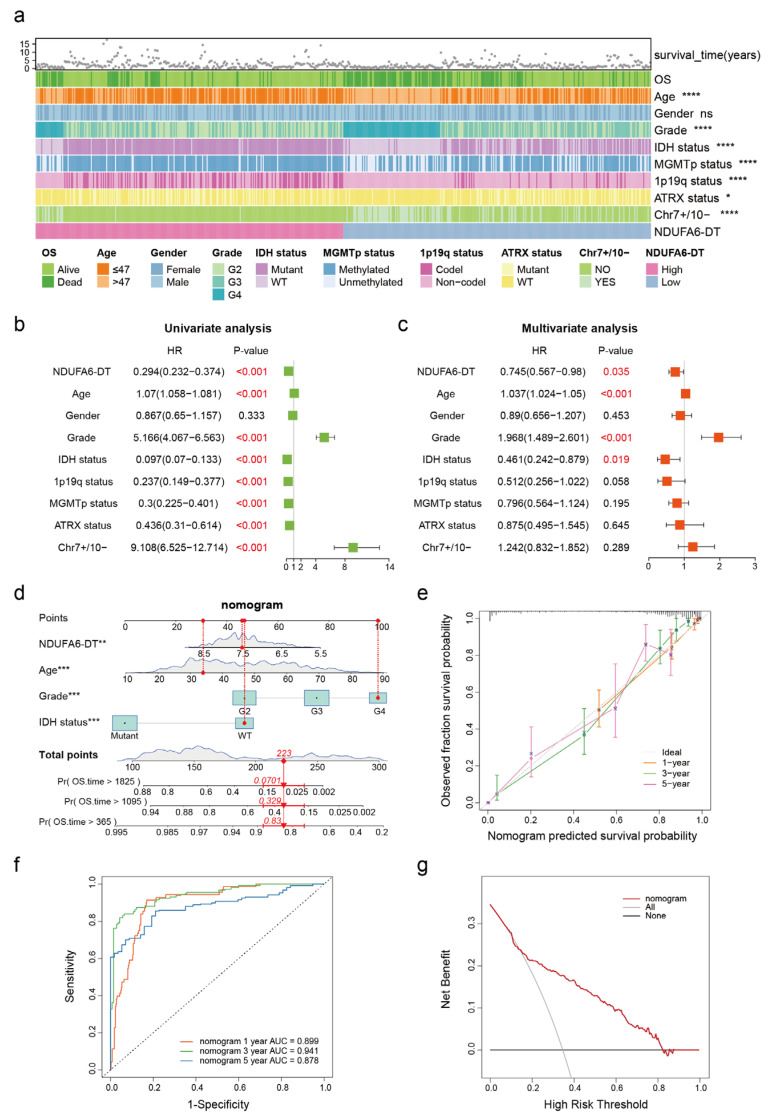
Regarding glioma, NDUFA6-DT serves as an independent prognostic and protective factor. (**a**) The heatmap illustrates the correlation between NDUFA6-DT expression and glioma clinical characteristics. (**b**,**c**) Forest plots show the results of univariate and multivariate analyses of NDUFA6-DT and the clinical characteristics of glioma. The red font indicates a variable with a value of *p* < 0.05. (**d**) Nomogram constructed based on NDUFA6-DT expression, age, disease grade, and IDH mutation status. The red arrows and numbers in the panel indicate that the patient with glioma is 33 years old, has a disease stage of G4, wild-type IDH mutation status, and NDUFA6-DT expression of 7.5. These variables correspond to 30, 100, 47, and 46 points, respectively, resulting in a total of 223 points. This prediction suggests that the one-, three-, and five-year survival probabilities for this patient would be 83%, 32.9%, and 7.01%, respectively. (**e**) Calibration curves of the nomogram at 1, 3, and 5 years. (**f**) ROC curves of the nomogram at 1, 3, and 5 years. (**g**) Decision curves for the nomogram. * *p* < 0.05, ** *p* < 0.01, *** *p* < 0.001, and **** *p* < 0.0001; ns, no significance.

The glioma samples were divided into high- and low-expression groups based on the expression of NDUFA6-DT to explore the correlation between NDUFA6-DT and clinical characteristics. Significant differences in NDUFA6-DT expression were observed across patient age, disease grading, IDH status, MGMTp status, 1p19q status, ATRX status, and the presence of chromosome 7 amplification (Chr7+) and chromosome 10 deletion (Chr10-), with no significant differences in sex (Figure 5a). The predictive significance of NDUFA6-DT and the clinical features of the gliomas were subsequently examined through multivariate and univariate analyses employing the Cox proportional hazards model. The findings revealed that NDUFA6-DT, age, disease grade, and IDH status were independent prognostic factors for gliomas (Figure 5b,c). Moreover, the hazard ratio (HR) for NDUFA6-DT was <1, suggesting a positive association between patient survival and gene expression levels. NDUFA6-DT operates as an independent glioma-protective factor.

Age, disease grade, IDH status, and NDUFA6-DT, which are the four independent prognostic indicators derived from the Cox regression analysis, were utilized to construct a nomogram that predicted overall survival at 1, 3, and 5 years, providing a visual representation of the predictive capacity of NDUFA6-DT in patients with gliomas (Figure 5d). The nomogram exhibited a C-index of 0.8632, suggesting robust predictive performance. Calibration curves at 1, 3, and 5 years illustrated a high level of agreement between the expected and true survival (Figure 5e). AUC values at 1, 3, and 5 years were 0.899, 0.941, and 0.878, respectively, suggesting that the nomogram exhibited satisfactory accuracy in predicting survival (Figure 5f). Based on the DCA (Figure 5g), the nomogram exhibited significant clinical value within a risk threshold range of 0.2–0.8, and similar results were observed during the analysis of the external validation cohort CGGA data (Figure S2). These findings emphasize the robust correlation between NDUFA6-DT and clinical characteristics, providing substantial support for glioma prognosis.

### 3.5. Potential Functions and Pathways of NDUFA6-DT in Gliomas

To elucidate the biological characteristics of NDUFA6-DT in glioma development, we employed differential expression and correlation analyses for the NDUFA6-DT high- and low-expression groups. Genes identified in the overlap of these analyses were designated as co-expressed NDUFA6-DT genes (Figure S3). These genes underwent GO, KEGG, and GSEA analyses.

**Figure 6:**
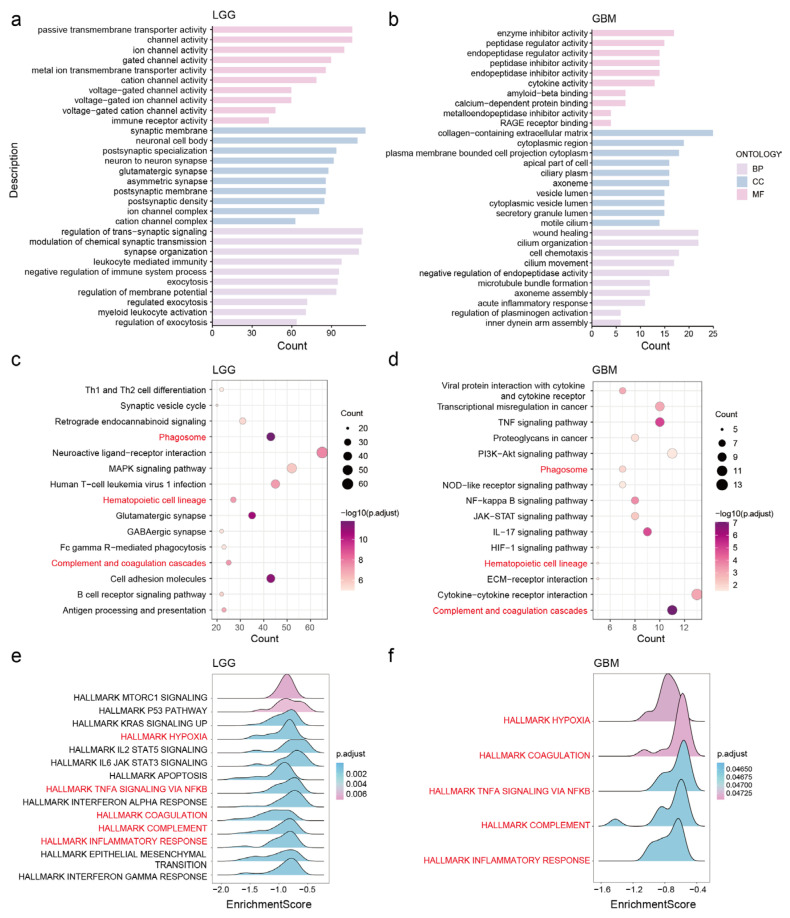
Elucidating the potential functions and signaling pathways of NDUFA6-DT in gliomas. (**a**,**b**) Bar graphs illustrate the GO analysis of NDUFA6-DT in LGG and GBM, emphasizing biological processes (BPs), cellular components (CCs), and molecular functions (MFs). (**c**,**d**) Bubble charts showing the results of the KEGG analysis of NDUFA6-DT in LGG and GBM. (**e**,**f**) Mountain plots illustrating GSEA results for NDUFA6-DT in LGG and GBM. The red font indicates that the same pathway is present in LGG and GBM.

GO analysis revealed that in LGG, NDUFA6-DT is associated with molecular functions encompassing ion channel activity, transmembrane transporter activity, and immune receptor activity. In terms of cellular composition, it is related to synaptic and channel complexes. Furthermore, it plays a role in biological processes including the regulation of trans-synaptic signaling, chemical synaptic transmission, exocytosis, and the immune system (Figure 6a). In GBM, NDUFA6-DT primarily exhibits molecular functions such as peptidases, endopeptidase regulator activity, and cytokine activity. It is localized within cellular components such as the vesicle lumen, collagen-containing extracellular matrix, and axoneme. Additionally, it participates in biological processes like cilium composition and movement, regulation of peptidase activity, cell chemotaxis, and inflammatory response (Figure 6b).

The KEGG enrichment analysis revealed that in LGG, NDUFA6-DT was associated with nervous system pathways, including neuroactive ligand–receptor interactions, glutamatergic synapses, and GABAergic synapses. It is also involved in cell adhesion molecules, phagosomes, and MAPK signaling pathways. Additionally, it plays a role in antigen processing and presentation as well as complement and coagulation cascades within the immune system (Figure 6c). In GBM, NDUFA6-DT primarily participated in the immune system regulation, including the complement and coagulation cascades and the IL-17 signaling pathway. It is also involved in cell signaling pathways, including the TNF, NF-kappa B, and PI3K-Akt signaling pathways, and cytokine–cytokine receptor interaction. Furthermore, NDUFA6-DT was associated with transcriptional dysregulation in cancer (Figure 6d).

Variations across NDUFA6-DT high and low expression levels were explored using GSEA, highlighting that the high-expression group of NDUFA6-DT did not exhibit enrichment outcomes in LGG and GBM. Conversely, the low-expression group of NDUFA6-DT in LGG demonstrated involvement in the immune response, hypoxia, proliferation, apoptosis, epithelial–mesenchymal transition, and signal transduction (Figure 6e). Similarly, the low-expression group of NDUFA6-DT in GBM was associated with immunological responses and hypoxic processes (Figure 6f). These findings suggest that NDUFA6-DT influences immunological responses, disrupts specific signaling pathways, and regulates synaptic transmission, influencing the onset and progression of gliomas.

### 3.6. The Expression of NDUFA6-DT Is Associated with Distinct Patterns of Immune Infiltration in Gliomas

**Figure 7:**
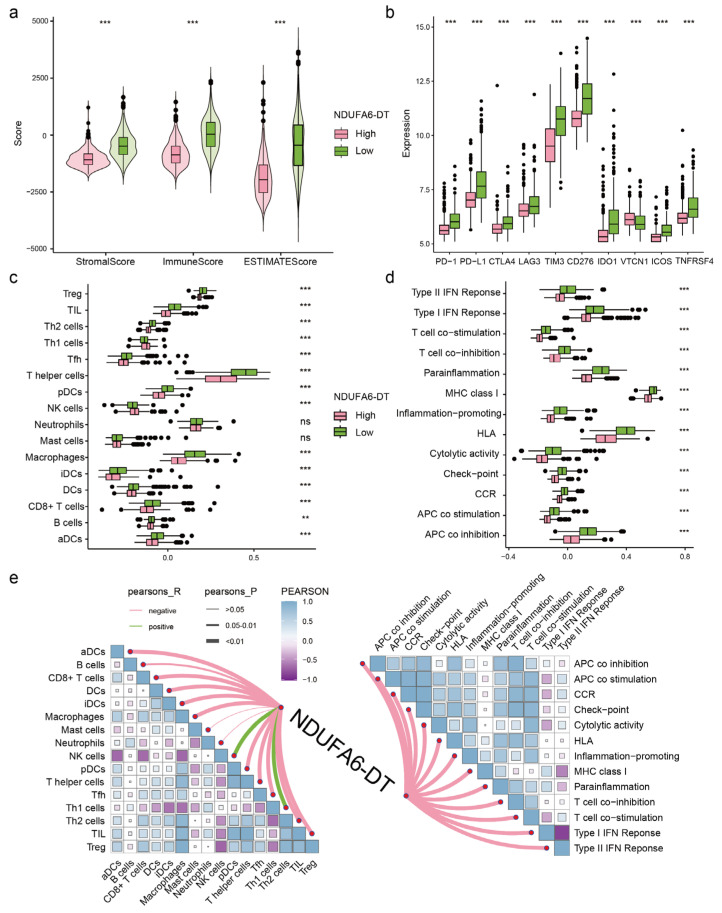
Exploring the relationship between NDUFA6-DT expression and immune infiltration. (**a**) Violin plots show differences in stromal scores, immunity scores, and estimated scores among NDUAF6-DT expression groups. (**b**–**d**) Box plots display differences in immune checkpoints, immune cells, and immune functions among NDUFA6-DT expression groups, respectively. (**e**) A correlation network diagram illustrates the relationship between NDUFA6-DT and immune cells as well as immune functions. ** *p* < 0.01, *** *p* < 0.001; ns, no significance.

Enrichment analysis showed that NDUFA6-DT was associated with immunity. Therefore, we further analyzed the relationship between NDUFA6-DT and immune infiltration. Estimation analysis revealed significantly higher stromal score and immunity score in the low-expression group compared to the high-expression group (Figure 7a). Additionally, the expression of immune checkpoints was higher in the low-expression group (Figure 7b). ssGSEA assessment demonstrated that the NDUFA6-DT high-expression group exhibited a greater degree of Th1 and NK cell infiltration, while all other 14 immune cells were significantly infiltrated in the low-expression group. Moreover, the low-expression group displayed more active immune function than the high-expression group (Figure 7c,d). Correlation analysis indicated that NDUFA6-DT expression was independent of mast cell and neutrophil infiltration but positively correlated with Th1 and NK cells. Conversely, it negatively correlated with other immune cells and immune functional activity (Figure 7e). These results reveal distinct patterns of immune infiltration in glioma samples based on NDUFA6-DT expression levels.

### 3.7. Potential ceRNA Network of NDUFA6-DT in Gliomas

**Figure 8:**
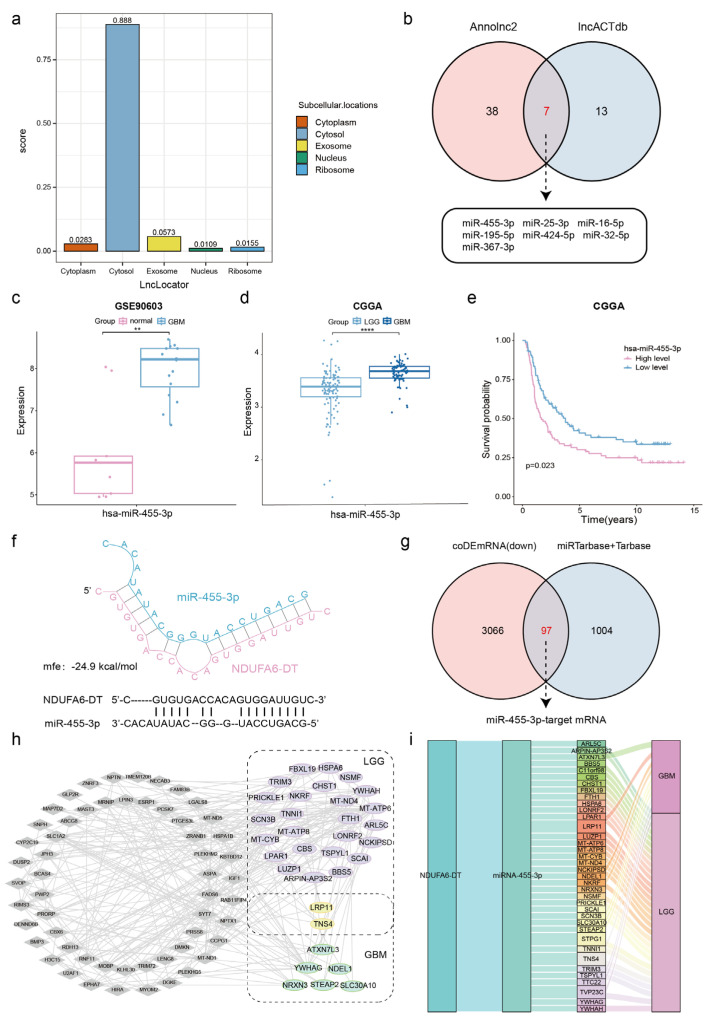
Exploring the potential ceRNA network of NDUFA6-DT in gliomas. (**a**) LncLocator predicts the subcellular localization of NDUFA6-DT. (**b**) The Venn diagram illustrates the seven overlapping miRNAs interacting with NDUFA6-DT in Annolnc2 (left) and lncACTdb (right). (**c**) Expression profile of miR-455-3p in normal and GBM samples from the GSE90603 dataset. (**d**) Expression levels of miR-455-3p in LGG and GBM from CGGA. (**e**) The Kaplan–Meier curve of miR-455-3p in CGGA. (**f**) RNAhybrid predicts the binding site of NDUFA6-DT with miR-455-3p. (**g**) Venn diagram illustrates the ninty-seven overlap of downregulated differential mRNAs in LGG and GBM (left) and the predicted target mRNAs of miR-455-3p from miRTarbase and Tarbase (right), representing the targeted mRNAs of miR-455-3p in gliomas. (**h**) The network graph displays differentially expressed mRNAs with survival significance selected from miR-455-3p-targeted mRNAs in gliomas. (**i**) The Sankey diagram displays the potential ceRNA network of NDUFA6-DT in gliomas. ** *p* < 0.01; **** *p* < 0.0001.

To explore the gene regulation mechanism of NDUFA6-DT in gliomas, we employed the ceRNA regulatory network, incorporating downregulated NDUFA6-DT, upregulated miRNAs, and downregulated mRNAs. Using LncLocator, we predicted the subcellular location of NDUFA6-DT, revealing the accumulation in the cytoplasm. This suggests its potential role as a ceRNA, interacting with miRNAs and serving as an miRNA sponge (Figure 8a).

Utilizing the AnnoLnc2 and lncACTdb databases, we predicted the interaction of seven miRNAs with NDUFA6-DT (Figure 8b). Validation of the expression of these miRNAs was conducted through analysis of the GSE90603 and GSE25632 datasets. The findings revealed significantly elevated levels of miR-455-3p, miR-424-5p, and miR-16-5p in patients with glioblastomas than in normal samples (Figure 8c and Figure S4a,b). Survival analysis conducted with CGGA data revealed the variations in the expression of four miRNAs, excluding miR-25-3p, miR-195-5p, and miR-367-3p (Figure 8e and Figure S4c–i). Conflicting expression profiles of miR-424-5p and miR-16-5p have been observed in gliomas [24,25,26]. However, consistent data corroborate a substantial upregulation of miR-455-3p [27,28,29]. Therefore, we hypothesize that NDUFA6-DT regulates functions as a sponge for miR-455-3p, thereby regulating downstream gene expression in gliomas. The CGGA data further suggest a significant elevation in miR-455-3p expression corresponding to the severity of the disease (Figure 8d).

Subsequently, the RNAhybrid tool was employed to predict the binding site between NDUFA6-DT and miR-455-3p, identifying a specific binding site within the region of NDUFA6-DT with an mfe of −24.9 kcal/mol (Figure 8f). The miRTarbase and Tarbase databases, repositories of experimentally confirmed miRNA–mRNA target relationships, were employed to determine the target mRNAs of miR-455-3p. By intersecting 1101 predicted mRNAs from these databases with 3163 mRNAs significantly downregulated in LGG and GBM, we identified 97 glioma-associated target mRNAs of miR-455-3p (Figure 8g). Survival analysis revealed 27 and 8 targeted mRNAs with prognostic significance in LGG and GBM, respectively (Figure 8h). The Sankey diagram illustrates the ceRNA network linked to NDUFA6-DT in LGG and GBM (Figure 8i). Thus, the NDUFA6-DT/miR-455-3p axis may represent a crucial ceRNA network in gliomas.

### 3.8. Potential Regulatory Mechanisms of the NDUFA6-DT-Associated ceRNA Network in Gliomas

**Figure 9:**
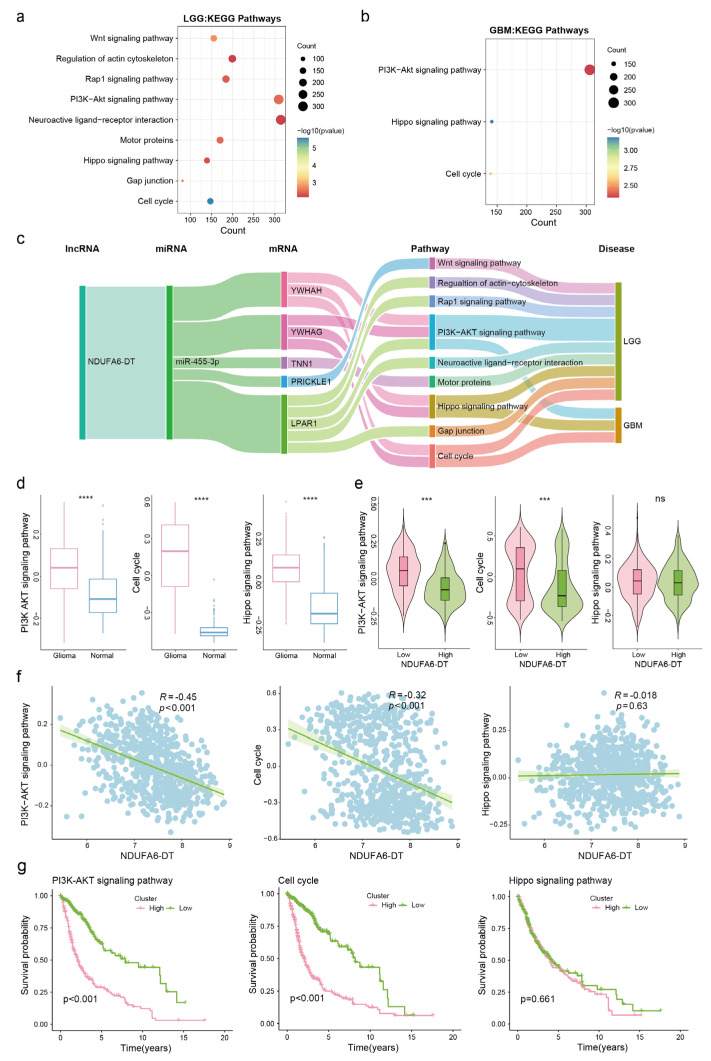
Exploring the potential regulatory mechanisms of the NDUFA6-DT-associated ceRNA network in glioma. (**a**,**b**) Bubble charts illustrate the KEGG pathway enrichment analysis for mRNAs regulated by NDUFA6-DT through miR-455-3p in LGG and GBM. (**c**) Network graphs display the ceRNA axes of NDUFA6-DT and the downstream signaling pathways regulated in LGG and GBM. (**d**) Box plots illustrate the differences in activity levels of the PI3K-AKT signaling pathway, cell cycle, and Hippo signaling pathway between normal and glioma samples. (**e**) Violin plots demonstrate the variations in activity levels of the PI3K-AKT signaling pathway, cell cycle, and Hippo signaling pathway between high- and low-expression groups of NDUFA-DT. (**f**) Scatter plot showing the correlation between NDUFA-DT expression and activity levels of the PI3K-AKT signaling pathway, cell cycle, and Hippo signaling pathway. (**g**) Survival curves for the PI3K-AKT signaling pathway, cell cycle, and Hippo signaling pathway. *** *p* < 0.001, **** *p* < 0.0001; ns, no significance.

To elucidate the potential pathways linked to the NDUFA6-DT-associated ceRNA network in gliomas, we employed DEGs for KEGG pathway enrichment, revealing the downstream signaling pathways. Based on the results of the KEGG enrichment analysis, the NDUFA6-DT-associated ceRNA network co-regulates the cell cycle, Hippo signaling system, and PI3K-Akt signaling pathway in LGG and GBM. Furthermore, in LGG, the network influenced glioma progression through the Rap1 and Wnt signaling pathways, neuroactive ligand–receptor interaction, and regulation of the actin cytoskeleton (Figure 9a,b). The visualization of potential regulatory mechanisms of NDUFA6-DT-associated ceRNAs in gliomas was achieved using the networkD3 package. NDUFA6-DT functioned as a sponge for miR-455-3p in LGG and GBM, regulating YWHAH and YWHAG, two members of the 14-3-3 protein family. This regulation affected the PI3K-Akt and Hippo signaling pathways and cell cycle (Figure 9c). The Pathview package was utilized to visualize the location and expression of 14-3-3 proteins in these pathways (Figure S5). The protein expression levels of YWHAG and YWHAH were examined in normal brains, LGGs, and GBMs using the HPA database. The results revealed a progressive decline in the expression levels of YWHAG and YWHAH with increasing disease grade, which exhibited a positive correlation with NDUFA6-DT (Figure S6). Furthermore, in LGG, NDUFA6-DT, through PRICKLE1, influences the Wnt signaling pathway and, through LPAR1, regulates neuroactive ligand–receptor interaction. These findings revealed the role of NDUFA6-DT in gliomas and its regulatory capacity over multiple crucial signaling pathways through its ceRNA network. These study findings provide initial and significant insights into the molecular mechanisms of gliomas.

The signaling pathways co-regulated by the NDUFA6-DT-associated CeRNA network in LGG and GBM were further analyzed. The activities of the PI3K-AKT signaling pathway, cell cycle, and Hippo signaling pathway were significantly increased in glioma patients compared to normal samples (Figure 9d). According to the expression grouping of NDUFA6-DT, we found that the activities of the PI3K-AKT and cell cycle pathways were significantly higher in the low-expression group than in the high-expression group, whereas there was no significant difference in Hippo signaling pathway activity (Figure 9e). The results of correlation and survival analyses showed a negative correlation between NDUFA6-DT expression and PI3K-AKT as well as cell cycle pathway activity. High pathway activity led to poor patient prognosis, while there was no association between the Hippo signaling pathway and NDUFA6-DT expression or patient prognosis (Figure 9f,g). These results suggest that low expression of NDUFA6-DT and enhanced activity of the PI3K-AKT signaling pathway and cell cycle contribute to a poor patient prognosis.

## 4. Discussion

LGG frequently progresses to a more aggressive form of GBM [4]. Integrating biomarker-targeted therapies enhances treatment efficacy and guides clinical decision making for gliomas [30]. The potential value of lncRNAs has been observed in diagnosing and treating gliomas [9,10].

This study found NDUFA6-DT to be a potential glioma biomarker. While research on NDUFA6-DT in tumors and gliomas is currently limited, its increasing recognition in the field of oncology is extensive. Previous studies have identified NDUFA6-DT as a prognostic lncRNA in breast [31,32,33] and thyroid cancers [34]. Functioning as a protective factor, it carries potential implications as a cancer prognostic indicator and therapeutic target. Li [35] and Niu [36] constructed a glioblastoma risk prediction model incorporating NDUFA6-DT and four other lncRNA signatures. They demonstrated its independent protective role through univariate and multivariate Cox regression analyses. Moreover, our results align with the findings of Li et al., suggesting that NDUFA6-DT expression is significantly higher in adjacent normal tissue than in GBM tissue. However, existing research on NDUFA6-DT predominantly concentrates on GBM, leaving its role in LGG and broader gliomas unknown. Our findings not only confirm the predictive utility of NDUFA6-DT in LGG but also provide insights into its biological mechanisms in gliomas.

Multidimensional analyses highlighted the significant clinical potential of NDUFA6-DT in gliomas. Initially, a comparison of NDUFA6-DT expression distributions with clinical characteristics revealed that low NDUFA6-DT expression is linked to poor prognosis. Moreover, it is particularly associated with high chr7+/chr10- alterations and a lower incidence of IDH mutations, MGMTp methylation, 1p19q co-deletion, and ATRX mutations, consistent with findings from existing research [37,38,39,40]. Second, exploring the association between NDUFA6-DT expression and genomic alterations revealed distinct mutational patterns that may potentially differ in their influence on TMB between LGG and GBM. Survival analysis, incorporating NDUFA6-DT expression and TMB status, further highlights the clinical significance of these genomic alterations. Finally, the prognosis of patients with gliomas can be accurately and effectively predicted by developing a nomogram that integrates the three clinical factors with NDUFA6-DT. These findings confirm that NDUFA6-DT is an independent prognostic factor with protective effects.

Co-expression analysis revealed that in LGG, NDUFA6-DT exhibits a positive correlation with PHLPP2 [41] and GRIA2 [42], known for inhibiting glioma progression. In contrast, it demonstrates a negative correlation with GMFG [43], S100A6, and S100A11 [44], all of which are associated with promoting glioma progression. Similarly, in GBM, NDUFA6-DT demonstrates a negative correlation with SUMF1 [45], CD109 [46], MUC11 [47], and MYBPH [48], which are all implicated in promoting glioma progression. These findings emphasize that NDUFA6-DT is linked to cancer suppression in gliomas. To the best of our knowledge, this is the first study to explore the role and function of NDUFA6-DT in gliomas. The distinct expression patterns of NDUFA6-DT in various clinical characteristics, LGG and GBM, suggest diverse biological functions. In LGG, NDUFA6-DT is linked to neurotransmitter release, synaptic organization, and immune system function. In GBM, it is associated with cellular structural organization, inflammation, and cell signaling pathways. GSEA reveals the participation of NDUFA6-DT low-expression groups in LGG and GBM in immune and hypoxic processes, highlighting the critical role of hypoxia and immune regulation in the brain tumor microenvironment [49,50]. NDUFA6-DT has been recognized as an immune-related lncRNA in sepsis [51], providing additional support for the implication of NDUFA6-DT in glioma progression. However, further experimental validation is necessary.

**Figure 10:**
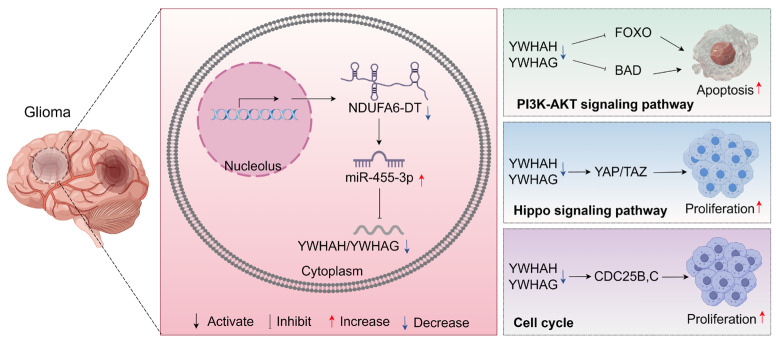
The mechanistic map illustrates the role of NDUFA6-DT as a sponge for miR-455-3p in gliomas, disrupting its ability to suppress YWHAH/YWHAG expression. This influences the regulation of the PI3K-AKT and HIPPO signaling pathways and the cell cycle, subsequently affecting tumor cell survival and apoptosis. Reduced NDUFA6-DT expression in disease states correlates with elevated tumor cell survival and apoptosis.

An essential step in investigating the functional pathways of long noncoding RNAs is identifying their targets. The construction of ceRNA networks not only enhances our comprehension of gene regulation and disease mechanisms but also provides novel avenues and strategies for disease treatment and prevention [52,53]. We hypothesized that NDUFA6-DT functions as a sponge for miR-455-3p, regulating several downstream mRNAs, including YWHAH, YWHAG, LPAR1, and others. Building upon this, we explored the shared regulatory mechanisms of NDUFA6-DT in LGG and GBM, with a particular focus on its indirect regulation of YWHAH and YWHAG. YWHAH and YWHAG are members of the highly conserved 14-3-3 protein family, consisting of seven members present in mammals (β, γ, ε, σ, ζ, τ, and η). These proteins play a regulatory role in various cellular processes, influencing survival and apoptotic signaling, cell proliferation, and cancer progression [54,55]. Our enrichment results revealed the participation of YWHAH (14-3-3γ) and YWHAG (14-3-3η) in the PI3K-AKT and Hippo signaling pathways and cell cycle in gliomas. Research suggests that these signaling pathways influence the prognosis of gliomas [56], with the PI3K-AKT pathway being recognized as a core signaling pathway in gliomas, especially in GBM [57]. Wang et al. reported that miR-217 promotes the growth and invasion of glioblastoma stem cells by inhibiting YWHAG. Consequently, YWHAG accelerates MDM4 phosphorylation, resulting in P53 degradation [58]. The concurrent decrease in 14-3-3η during radiotherapy heightens GBM sensitivity to radiation [59]. Yao et al. discovered that Comp 5 (an SIRT1 activator) triggers autophagy by downregulating 14-3-3γ [60]. In summary, we propose the potential existence of an NDUFA6-DT-miR-3p-YWHAH/YWHAG axis regulating the PI3K-AKT signaling pathway and thereby influencing glioma development in LGG and GBM (Figure 10).

Our study had some limitations. First, our conclusions rely on public databases, which are derived from bioinformatics algorithms owing to the inherent constraints of data sources and research techniques. While these algorithms may not comprehensively capture the relevant biological mechanisms, they provide valuable guidance. Second, the retrospective nature of survival analysis data may have limitations in timeliness, as real-time updates and enhancements in data quality are lacking. Finally, additional in vitro and in vivo studies are required despite certain results being validated using external datasets. We maintain that our comprehensive algorithmic exploration and assessment of glioma gene expression data will provide crucial directions and reference points for future studies and medical procedures.

## 5. Conclusions

In conclusion, our study identified the lncRNA signature NDUFA6-DT as applicable to patients with gliomas across all grades. NDUFA6-DT exhibited a significant decrease in gliomas and was recognized as an independent protective factor. We assessed the prognostic and biomarker capabilities of NDUFA6-DT across various molecular subtypes of gliomas, offering potentially new perspectives for their diagnosis and treatment. Furthermore, this study represents the first to assess the role and functionality of NDUFA6-DT in gliomas, including its associated ceRNA regulatory network. NDUFA6-DT may play a crucial role in synaptic transmission and immunological responses in gliomas. Specifically, the NDUFA6-DT-miR-455-3p-YWHAH/YWHAG axis may regulate the PI3K-Akt signaling pathway, influencing glioma development. However, further experimental and clinical validations are necessary to confirm the findings of this study.

## Figures and Tables

**Table 1:** An overview of the datasets.

Accession/Cohort	Database	RNA Library	Sample Size
TCGA-LGG	TCGA	RNA sequencing	LGG: 528
TCGA-GBM	TCGA	RNA sequencing	GBM: 168
GTEx	GTEx	RNA sequencing	Normal brain: 289
mRNAseq_693	CGGA	RNA sequencing	LGG: 443, GBM: 249
mRNAseq_325	CGGA	RNA sequencing	LGG: 182, GBM: 139
GSE108474	GEO	RNA sequencing	Normal: 28, LGG: 215, GBM: 228
GSE90603	GEO	miRNA sequencing	Normal: 9, GBM: 16
GSE25631	GEO	miRNA sequencing	Normal: 5, GBM: 82

## Data Availability

The datasets presented in this study can be found in online repositories. The names of the repository/repositories and accession number(s) can be found in the Materials and Methods.

## Supplementary Materials

The following supporting information can be downloaded at: https://www.mdpi.com/article/10.3390/genes15040483/s1, Figure S1: Validation and selection of key lncRNAs in gliomas. Figure S2: CGGA as an external validation dataset for constructing the nomogram. Figure S3: Co-expressed genes of NDUFA6-DT in gliomas. Figure S4: Validation and selection of miRNAs interacting with NDUFA6-DT in gliomas. Figure S5: Localization and expression levels of NDUFA6-DT-targeted mRNAs in the PI3K-AKT signaling pathway, Hippo signaling pathway, and cell cycle. Figure S6: IHC staining plots of NDUFA6-DT-regulated downstream molecules YWHAG and YWHAH in normal brain, LGG and GBM from the HPA database. Figure S7: Sequence information of NDUFA6-DT.
