# Supplementary material for: LncRNA NDUFA6-DT: A Comprehensive Analysis of a Potential LncRNA Biomarker and Its Regulatory Mechanisms in Gliomas
Source: Genes (Basel). 2024 Apr 11;15(4):483. doi: 10.3390/genes15040483 (PMC11050413; doi:10.3390/genes15040483)
Supplement: Supplementary file 1 [file genes-15-00483-s001.zip › genes-2943378-supplementary.pdf]

genes

MDPI

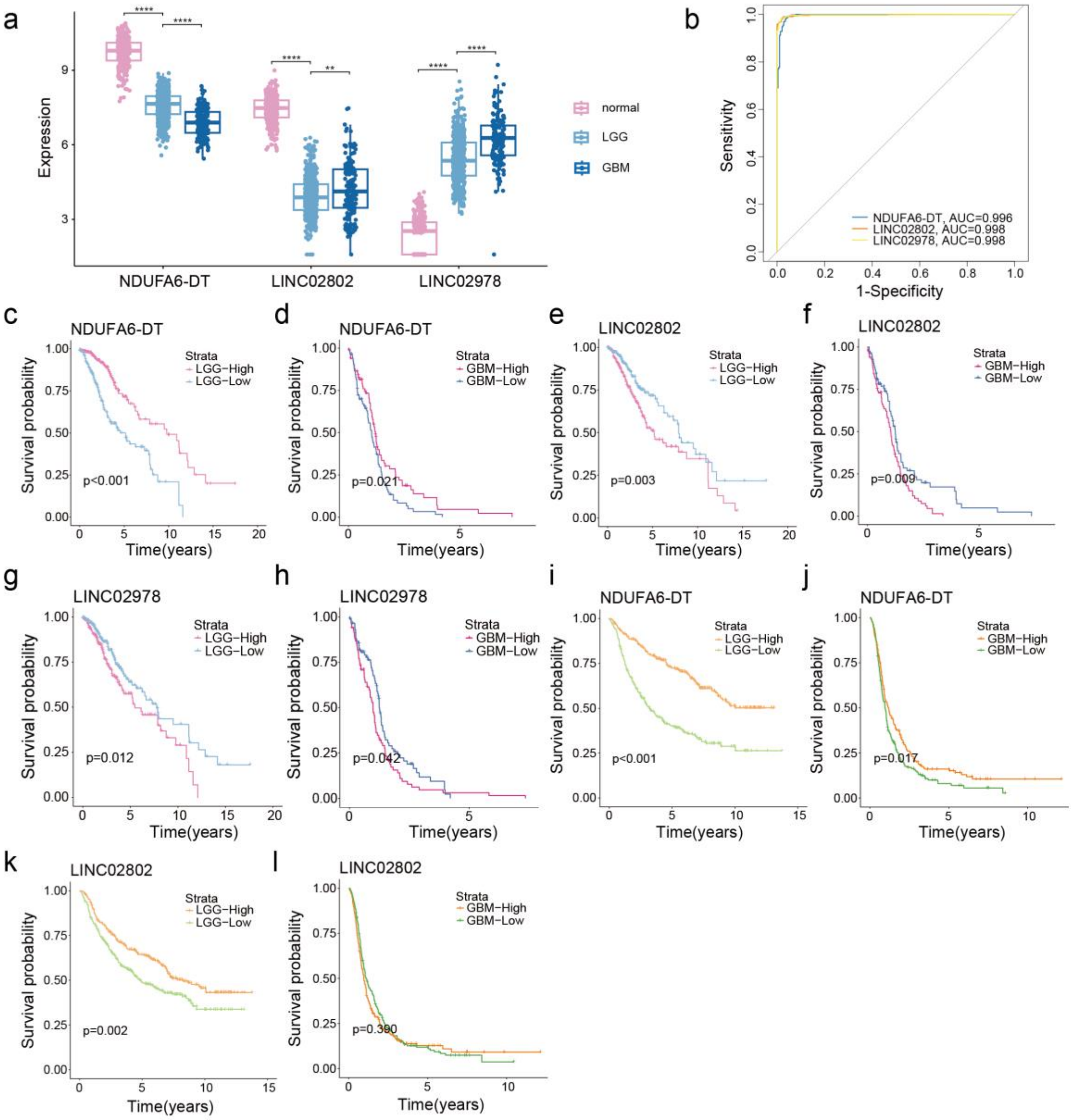

Figure S1. Validation and selection of key lncRNAs in gliomas. **(a)** Expression profiles of three lncRNAs in normal samples as well as in cases of both lower-grade glioma (LGG) and glioblastoma (GBM). **(b)** ROC curves of three lncRNAs in gliomas. **(c-h)** Kaplan-Meier curves for patients with high and low expression of NDUFA6-DT, LINC02802, and LINC02978 in LGG and GBM in the TCGA dataset. **(i-l)** Kaplan-Meier curves for patients with high and low expression of NDUFA6-DT and LINC02802 in LGG and GBM in the CGGA dataset. ***p* < 0.01, *****p* < 0.0001.

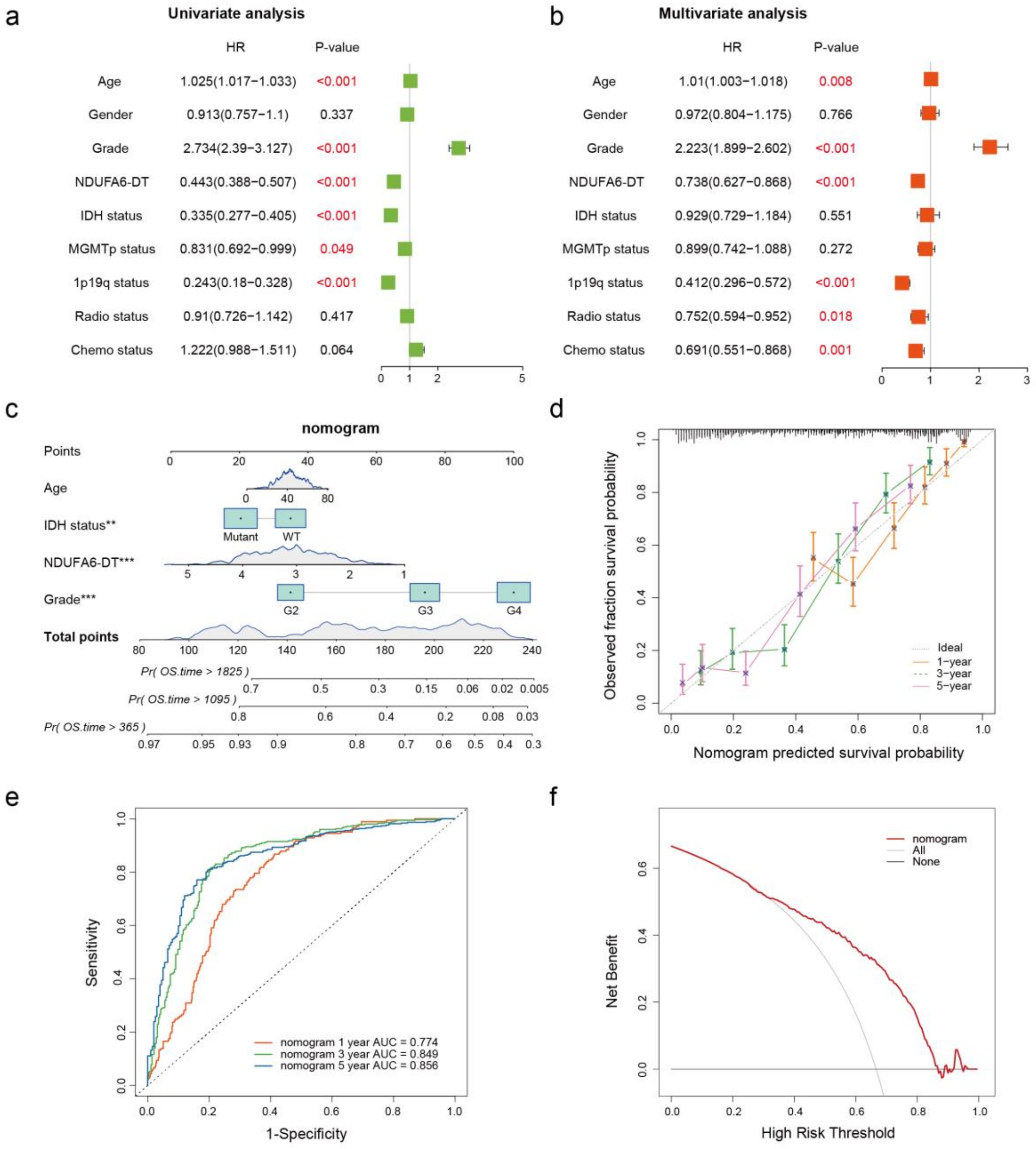

Figure S2. CGGA as an external validation dataset for constructing the nomogram. **(a-b)** Forest plots depicting univariate and multivariate Cox regression analyses of NDUFA6-DT with clinical characteristics in glioma. The red font indicates a variable with a value of $p < 0.05$. **(c)** A nomogram was constructed based on age, IDH mutation status, NDUFA6-DT expression, and disease grade. **(d)** Calibration curves for the nomogram were plotted at 1, 3, and 5 years. **(e)** ROC curves for the nomogram were generated at 1, 3, and 5 years. **(f)** The Decision curves for the nomogram. $^{**}p < 0.01$, $^{***}p < 0.001$.

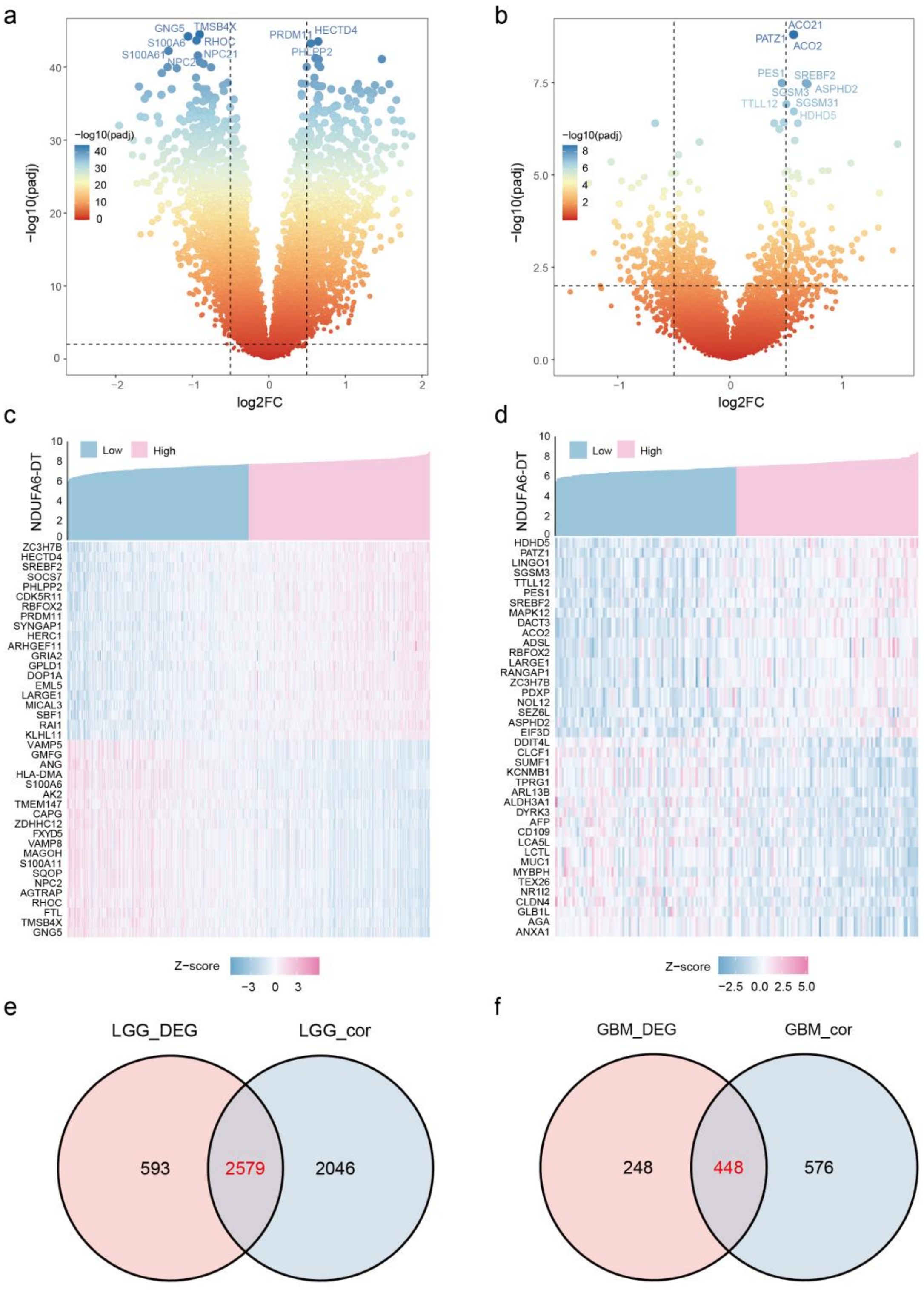

Figure S3. Co-expressed genes of NDUFA6-DT in gliomas. **(a-b)** Volcano plots depict differential expression analysis in high and low expression groups of NDUFA6-DT in LGG and GBM. **(c-d)** Heatmaps illustrate correlation analysis of NDUFA6-DT in LGG and GBM, with the top 20 genes positively correlated and the bottom 20 genes negatively correlated. **(e-f)** Venn diagrams show the intersection of differentially expressed and correlated genes as co-expressed genes with NDUFA6-DT in LGG and GBM.

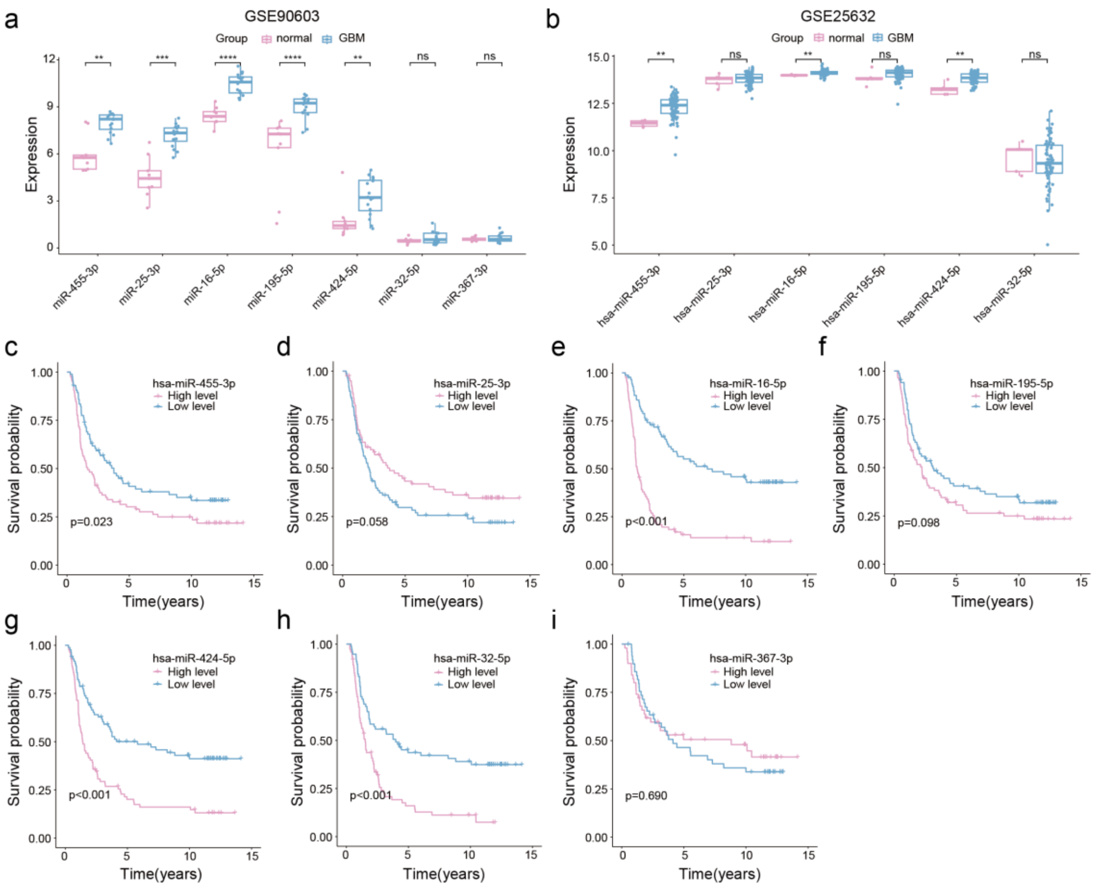

Figure S4. Validation and selection of miRNAs interacting with NDUFA6-DT in gliomas. **(a)** Expression profiles of 7 miRNAs in GSE90603 in normal samples and GBM. **(b)** Expression profiles of six miRNAs in GSE25632 in normal samples and GBM. **(c-i)** Kaplan-Meier curves for patients with high and low expression of miR-455-3p, miR-25-3p, miR-16-5p, miR-195-5p, miR-424-5p, and miR-367-3p in CGGA. ***p* < 0.01, ****p* < 0.001, and *****p* < 0.0001; ns, no significance.

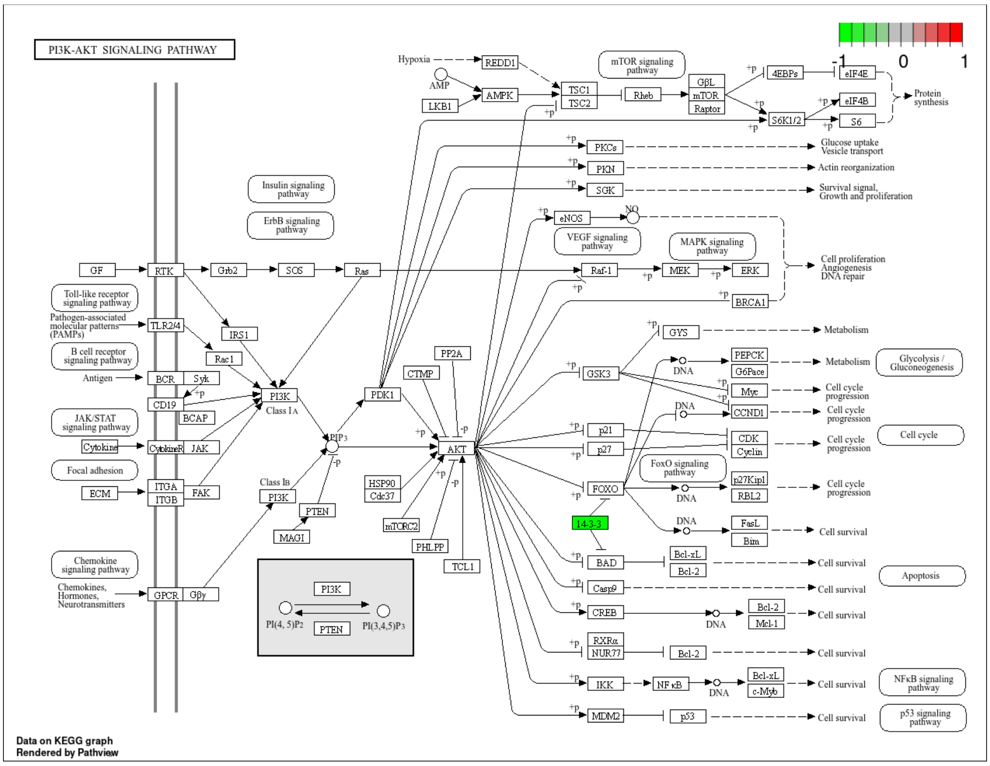

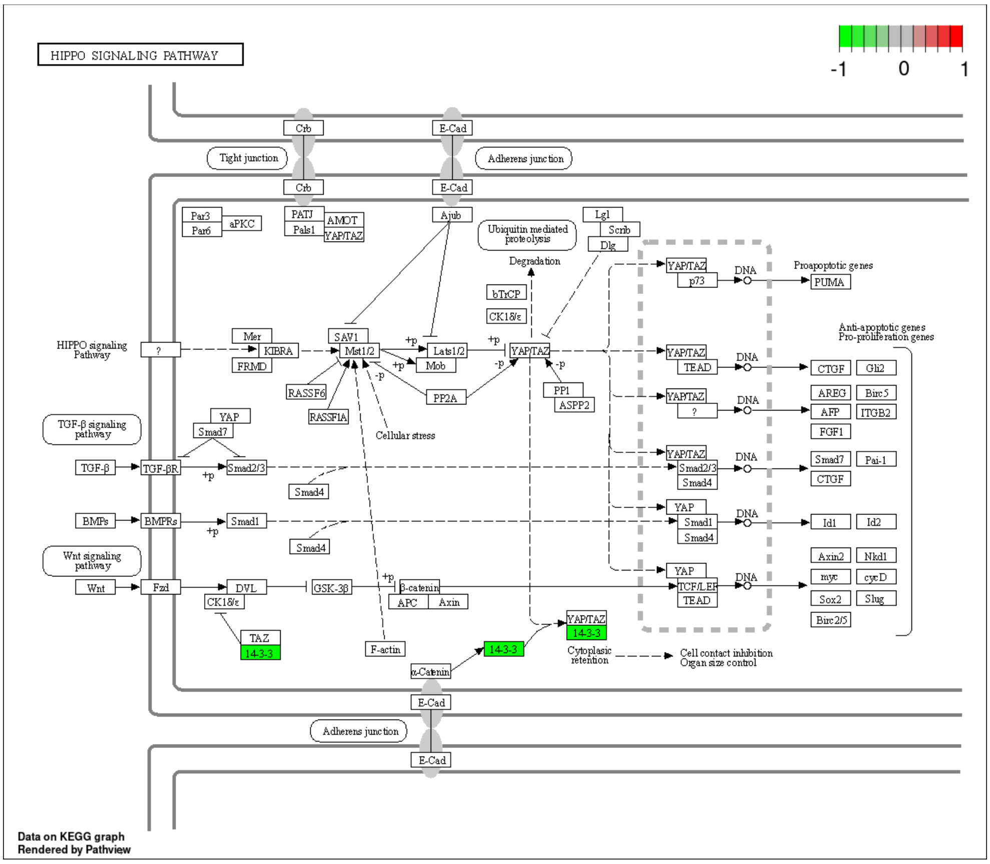

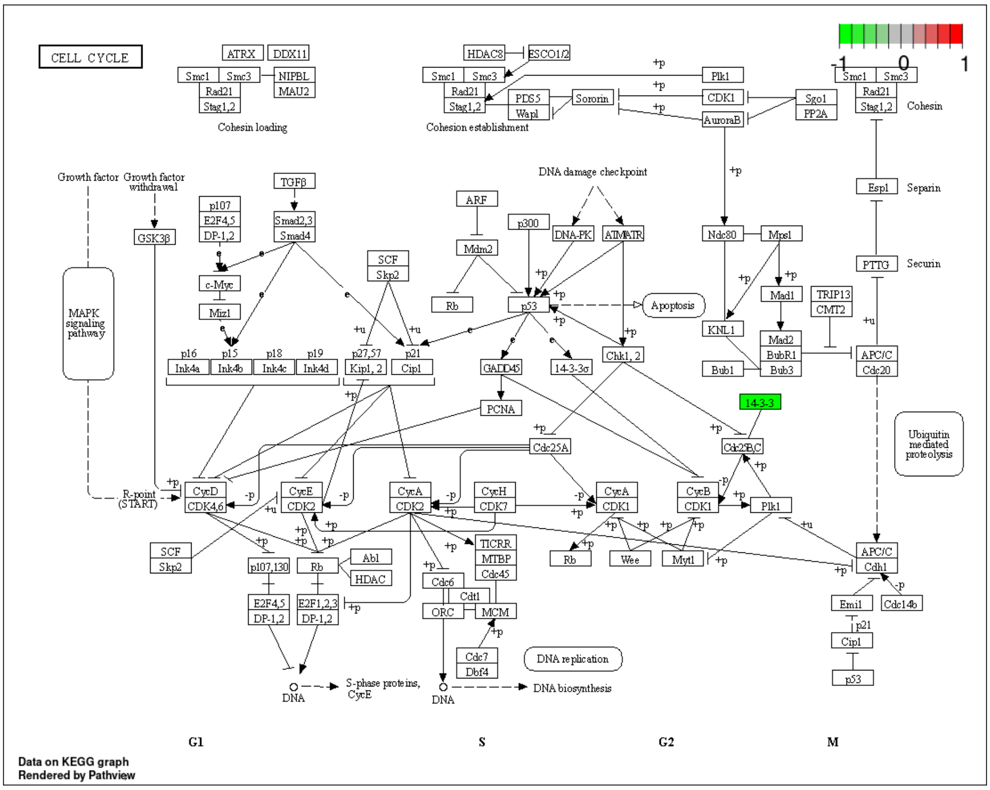

Figure S5. Localization and expression levels of NDUFA6-DT-targeted mRNAs in the PI3K-AKT signaling pathway, Hippo signaling pathway, and cell cycle. The red label indicates upregulation, while the green label represents downregulation.

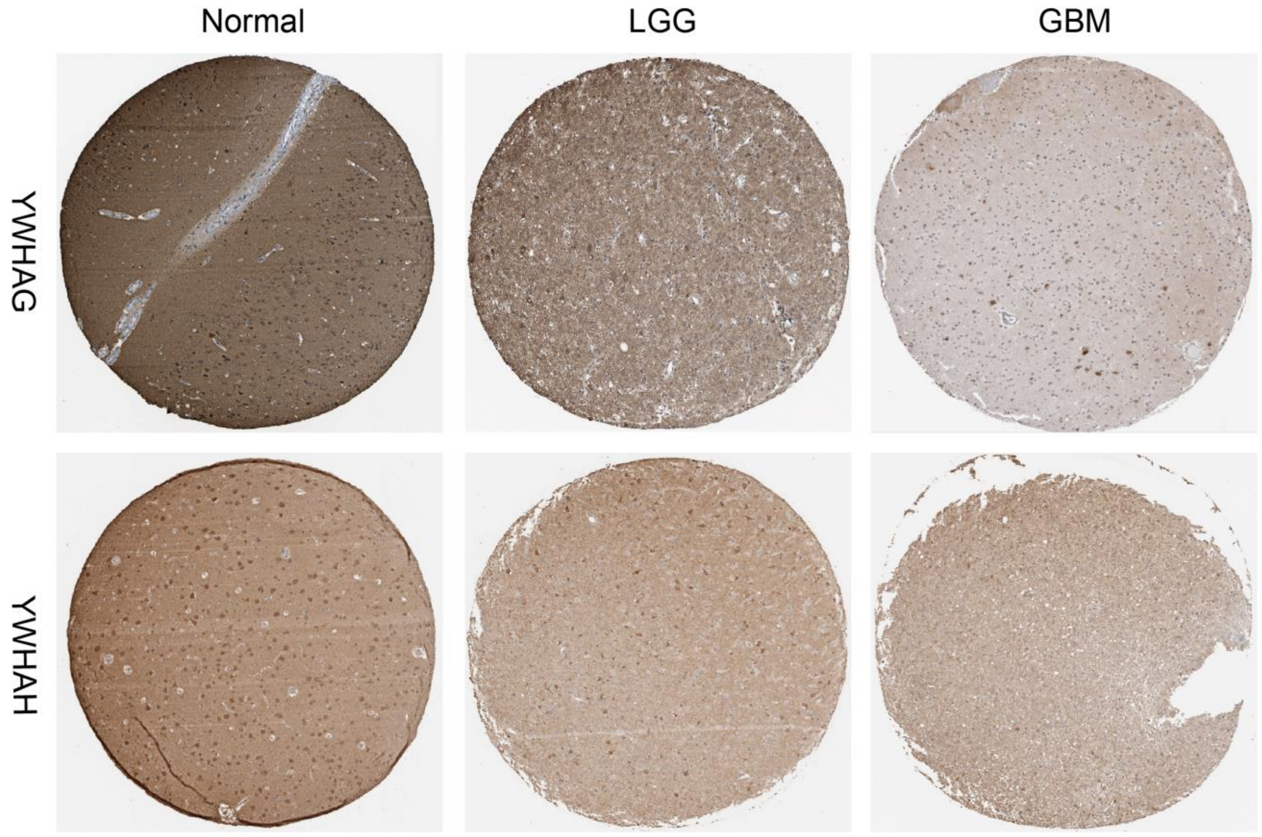

Figure S6. IHC staining plots of NDUFA6-DT-regulated downstream molecules YWHAG and YWHAH in normal brain, LGG and GBM from the HPA database.

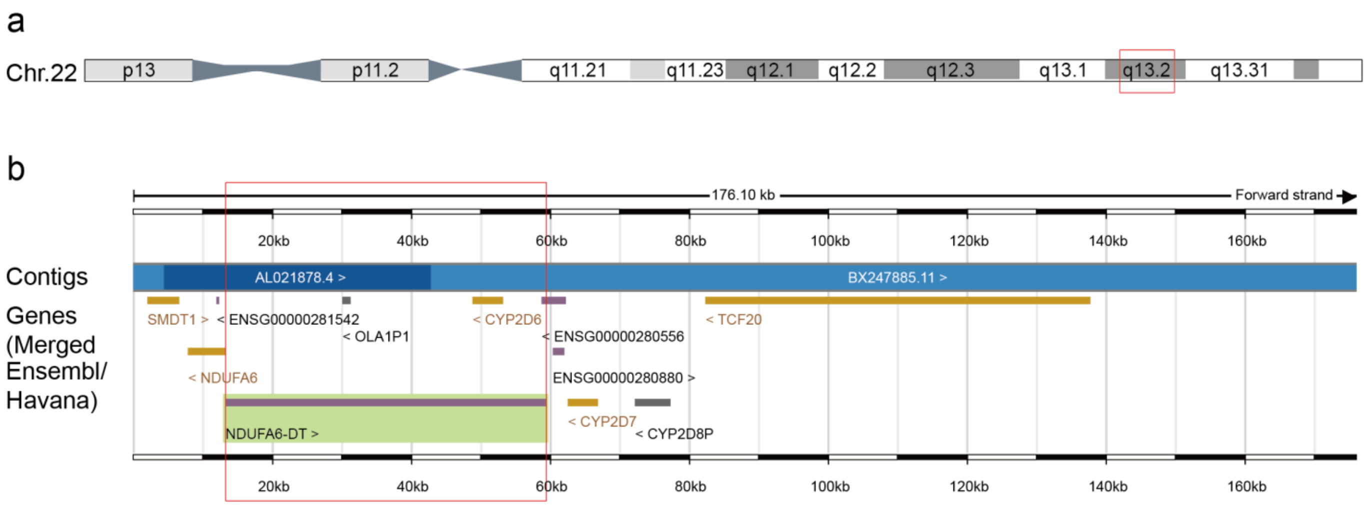

Figure S7. Sequence information of NDUFA6-DT. **(a)** Genomic location of NDUFA6-DT. **(b)** Length of NDUFA6-DT and nearby genes.
